# Bloodstream infections due to multi-drug resistant bacteria in the emergency department: prevalence, risk factors and outcomes—a retrospective observational study

**DOI:** 10.1007/s11739-024-03692-7

**Published:** 2024-07-13

**Authors:** Nicolò Capsoni, Giulia Maria Azin, Marida Scarnera, Marco Bettina, Riccardo Breviario, Laura Ferrari, Camilla Ferrari, Daniele Privitera, Chiara Vismara, Alessandra Bielli, Filippo Galbiati, Davide Paolo Bernasconi, Marco Merli, Michele Bombelli

**Affiliations:** 1 School of Medicine and Surgery, University of Milan-Bicocca, Milan, Italy; 2 Department of Emergency Medicine, ASST Grande Ospedale Metropolitano Niguarda, Milan, Italy; 3 Department of Medicine and Surgery, University of Milan, Milan, Italy; 4 Division of Infectious Diseases, ASST Grande Ospedale Metropolitano Niguarda, Milan, Italy; 5 Department of Clinical Research and Innovation, ASST Grande Ospedale Metropolitano Niguarda, Milan, Italy; 6 Chemico-Clinical and Microbiological Analyses, ASST Grande Ospedale Metropolitano Niguarda, Milan, Italy; 7 Internal Medicine, Pio XI Hospital, ASST Brianza, Desio, Italy

**Keywords:** Sepsis, Septic shock, MRSA, Beta-lactamases, *Enterobacteriaceae*, Anti-bacterial agents

## Abstract

Multidrug-resistant organisms (MDROs) are prevalent in patients admitted to the Emergency Department (ED) and increase the risk of inappropriate empirical antibiotic therapy. Risk stratification for MDRO infection is essential to early identify patients requiring empirical broad-spectrum antibiotic therapy, but it remains challenging for emergency physicians. This study aimed to evaluate prevalence, risk factors, and outcomes of patients admitted to the ED with a bloodstream infection (BSI) caused by MDROs. A retrospective observational study enrolling all consecutive adult patients admitted with a BSI to the ED of Niguarda Hospital, Italy, from January 2019 to December 2021 was performed. 757 patients were enrolled, 14.1% with septic shock. 156 (20%) patients had a BSI caused by MDRO: extended-spectrum beta-lactamase (ESBL) producing *Enterobacterales* were the most prevalent followed by methicillin-resistant *Staphylococcus aureus* (MRSA). Risk factors for BSI due to MDRO and specifically for ESBL were chronic renal failure (OR 2.2; 95%CI 1.4–3.6), nursing home residency (OR 4.4; 95%CI 1.9–10.2) and antibiotic therapy in the last 90–days (OR 2.6; 95%CI 1.7–4), whereas for MRSA were dialysis (OR 12.3; 95%CI 1.8–83), antibiotic therapy and/or hospital admission in the past 90-days (OR 3.6; 95%CI 1.2–10.6) and ureteral stent or nephrostomy (OR 7.8; 95%CI 1.5–40.9). Patients with BSI due to MDRO had a higher rate of inappropriate empirical antibiotic therapy (50%) and longer length of stay, but no higher in-hospital mortality. Among patients admitted to the ED with a BSI, MDROs are frequent and often associated with inappropriate empirical antibiotic therapy. Specific updated risk factors for MDRO may help clinicians to better identify patients requiring a broader antibiotic therapy in the ED, while awaiting microbiological results.

**Supplementary Information:**

The online version contains supplementary material available at 10.1007/s11739-024-03692-7.

## Introduction

Multidrug-resistant organisms (MDROs) have continuously spread over several years, and the prevalence of infection due to MDRO among patients presenting to the Emergency Department (ED) has increased [1–3]. Previous medicalization and exposure to the healthcare systems are frequent and represent major causes of this phenomenon. Indeed, patients admitted to the ED are aging, suffer from multiple comorbidities, and have often a history of repeated antibiotic treatments, frequent hospital admissions or day hospital visits, chronic immunosuppressive therapies, and invasive devices such as urinary or intravascular catheters [4–7].

The elevated prevalence of MDRO infection warrants careful consideration in the choice of empirical antibiotic treatment. In previous studies, the rate of inappropriate empirical antibiotic prescription was reported to be higher in patients with MDRO infections, reaching up to 50% and leading to increased mortality, especially in patients with sepsis and septic shock [8–11]. The choice of empirical antibiotic therapy depends on the origin and severity of the infection, local epidemiology, and evaluation of risk factors for MDRO [12].

Identifying patients with an increased risk of infection from multi-drug-resistant pathogens that necessitates broadening the antibiotic therapy spectrum is imperative for emergency physicians for two key reasons: to prevent ineffective initial treatment which may result in increased mortality and to limit the overuse of wide-spectrum antibiotics in low-risk individuals which may further increase both antimicrobial resistance and avoidable costs. On the other hand, to date, accurately stratifying the risk for MDRO infection and identifying patients who require a broader spectrum antibiotic therapy when admitted to the ED for infection remains challenging. Previously, a definition of healthcare-associated bloodstream infection (BSI) was proposed to identify patients who needed treatment with broad-spectrum antibiotics to cover for MDRO, but it subsequently proved to be of little help, resulting in the unnecessary use of second-line antibiotics, especially in settings where multidrug resistance is low [13, 14]. As many risk factors linked to MDRO infection have been reported, and a high proportion of patients present with at least one of them, they often lack of specificity and should be weighted more appropriately [4, 6, 7].

Continuous updating of the local epidemiology and reporting the most common risk factors associated with MDRO infection is essential to enhance appropriate empirical antibiotic therapy. While various studies have described the risk factors associated with MDRO infection in specific organ sites or pathogens, few have focused on BSI in the ED [15–22].

This study aimed to assess the prevalence, risk factors, and outcomes of patients admitted to the ED with BSI due to multi-drug-resistant bacteria.

## Methods

### Study design

A single-center retrospective observational cohort study enrolling all consecutive adult patients with bloodstream infection coming from the community and admitted to the ED of Niguarda Hospital, Milan, Italy, from January 2019 to December 2021 was conducted. The study was approved by the local ethical committee of Milano Area 3 (ethical approval number 338–18,052,022). Owing to retrospective and de-identified data collection, the need for informed consent was waived.

### Participants

All adult patients (≥ 18 years) who presented to the ED with a BSI were enrolled if the blood culture was performed < 48 h from hospital arrival. BSI was defined as a positive blood culture in a patient with systemic signs of infection and could be either primary (*i.e.* without an identified origin) or secondary to a documented source of infection [23]. In case of repeated admissions to the ED, patients were enrolled only once and data from the first access to the ED were included in the analysis.

Patients admitted to the ED from hospital wards or discharged within 48 h were excluded. Patients with contaminated blood cultures, defined as one or more microrganisms, as coagulase-negative staphylococci, *Micrococcus s*pp, Viridans group streptococci, *Cutibacterium acnes*, *Corynebacterium s*pp or *Bacillus*, present only in a single bottle or in a single set out of 2–3 sets of blood cultures, were excluded. Every case of suspected contaminant was evaluated by a panelist of emergency physicians, microbiologists, and infectious disease specialists [24].

### Data collection

All positive blood cultures collected from adult patients in the ED were obtained from the hospital microbiology laboratory database. The patient’s medical records were used to gather data, including demographics, comorbidities, risk factors for multidrug-resistant organisms (MDRO), Charlson Comorbidity Index, microbiological findings, infection location, disease severity, and empiric antibiotic therapy [25].

Risk factors potentially associated with MDRO included nursing home or long term care facilities (LTCF) residency, hospitalization for 2 or more days in the previous 90 days, antimicrobial therapy within the last 90 days, attendance of day-hospital wards within the last 90 days; chronic wound care, chronic dialysis, home intravenous therapy within the preceding 30 days, and the presence of indwelling bladder catheter, indwelling intravascular devices (*i.e.* peripheral vein catheter (PVC), central vein catheter (CVC), peripherally inserted central catheter (PICC, Porth-a-cath)), indwelling nephrostomy tube, biliary stent, and nasogastric/nasojejunal tube; severe immunosuppression (defined as the presence of at least one of the following medical conditions: active hematological malignancy, solid organ transplantation within 1 year, active immunosuppressive therapy, chemotherapy or radiotherapy within 30 days prior to their admission to the ED); mild-to-moderate immunodepression (defined by the presence of at least one of the following medical conditions: chronic systemic steroid therapy. *i.e*. prednisone ≥ 5 mg daily or equivalent, active solid malignancy, splenectomy, and autoimmune disease) [26].

This information was collected on a pre-filled Excel data set by physicians and residents of the ED.

### Microbiological data and empiric antimicrobial therapy

Identification of the isolates and susceptibility test were performed by Matrix Assisted Laser Desorption Ionization – Time of Flight (MALDI-TOF, Bruker Daltonics) and MicroScan WalkAway (Beckman Coulter), respectively. Susceptibility break-points were based on Anti-microbial Susceptibility Testing (EUCAST) updated guidelines.

Methicillin-resistant *Staphylococcus aureus* (MRSA), *Pseudomonas aeruginosa* resistant to three classes of antibiotics among antipseudomonal penicillins, anti-pseudomonal cephalosporins, carbapenems, quinolones, and aminoglycosides, vancomycin-resistant *Enterococcus*, carbapenem-resistant *Acinetobacter baumannii*, extended spectrum beta-lactamase (ESBL)-producing *Enterobacterales*, carbapenem-resistant *Enterobacterales* were considered as MDRO, as previously defined [27]. The ESBL phenotype was inferred based on resistance to third-generation cephalosporins.

Empiric antibiotic therapy administered in the ED was recorded, together with its appropriateness according to the in vitro antibiotic susceptibility of the isolated pathogen. The EUCAST breakpoints in force in the years of the study period were considered.

### Study definitions and outcomes

The severity of the infection was assessed through the acute change in total Sequential Organ Failure Assessment (SOFA) score at the time of ED presentation [28]. *Sepsis* was defined as acute SOFA score ≥ 2. *Septic shock* was defined as persisting hypotension requiring vasopressors to maintain mean arterial pressure ≥ 65 mmHg and having a serum lactate level > 2 mmol/L (18 mg/dL) despite adequate volume resuscitation [29].

The primary endpoint of the study was the evaluation of prevalence and risk factors for BSI due to MDRO. The secondary endpoints were the appropriateness of empiric antibiotic therapy prescription in the ED, in-hospital mortality, and length of hospital stay (LOS).

*In-hospital mortality* was defined as all-cause mortality that occurred during hospitalization.

The *length of stay (LOS)* referred to the number of days from hospital admission to discharge.

### Statistical analysis

We determined a sample size of 800 positive bloodstream cultures, assuming a 20% prevalence of MDROs (Multidrug-Resistant Organisms) based on prior estimate. The sample size was calculated to achieve a target precision in the estimation of the prevalence of MDRO BSI in the ED, indicated by a semi-amplitude of the 95% confidence interval (CI) of 3%.

The proportion of patients with MDRO BSI was calculated together with the 95% CI. Categorical variables were presented as absolute numbers and percentages. Continuous variables were described using either mean and standard deviation or, if the variable did not follow a normal distribution, median and interquartile range. For categorical variables, the Chi-square test and Fisher’s test were performed to assess differences between group, specifically comparing MDRO-positive patients vs non-MDRO-positive patients. For continuous variables, either Student’s t-test or the Wilcoxon–Mann–Whitney test were used, depending on whether the parameter followed a normal distribution. Similar methods were applied to evaluate differences between sub-groups, such as MRSA vs methicillin-sensitive *Staphylococcus aureus* (MSSA) BSI and ESBL-producing *Enterobacterales* vs non-ESBL-producing *Enterobacterales* BSI, etc.

Univariate and multivariate logistic regression analysis was performed to identify risk factors associated with MDRO BSI in general, as well as specifically for MRSA and ESBL-producing *Enterobacterales*. Finally, logistic regression was used to analyze the association between MDRO phenotype, as ESBL and MRSA, the appropriateness of empirical antimicrobial therapy, and other potential clinical risk factors with in-hospital mortality (Table 1).

**Table 1:** Study population

Total	757 (100)
*Demographics characteristics*	
Female, *n* (%)	292 (38.6)
Age, years, median (IQR)	71 (59–80)
*Comorbidities*, *n* (%)	
Charlson comorbidity index, median (IQR)	5 (3–7)
Hypertension	414 (54.7)
Diabetes mellitus	226 (29.9)
Chronic renal failure	170 (22.5)
Solid Cancer	156 (20.6)
Ischemic heart disease	95 (12.5)
Chronic obstructive pulmonary disease	92 (12.2)
Chronic heart failure	69 (9.1)
Hematological malignancy	65 (8.6)
Solid organ transplantation	61 (8.1)
Dementia	52 (6.9)
Chronic liver disease	49 (6.5)
Stroke	48 (6.3)
Bronchiectasis	16 (2.1)
Bone marrow transplantation	16 (2.1)
HIV infection	14 (1.8)
*Risk factors for MDRO infectious*, *n* (%)	
Antibiotic therapy in the last 90 days	326 (43.1)
Hospital admission in the last 90 days	307 (40.6)
Day hospital admission in the last 90 days	175 (23.1)
Chemotherapy in the last 30 days	101 (13.3)
Endovascular devices	94 (12.4)
Immunosuppressive therapy	74 (9.8)
Ulcers and difficult wounds	61 (8.1)
Permanent urethral catheter	58 (7.7)
Biliary stent	57 (7.5)
Chronic corticosteroid therapy	45 (5.9)
Ureteral stent	29 (3.8)
Nephrostomy	22 (2.9)
Dialysis	19 (2.5)
Nursing home or LTCF residency	28 (3.7)
Intestinal stoma	12 (1.6)
Nasogastric tube or EPG	12 (1.6)
Home parenteral therapy	7 (0.9)
ESBL producers colonization	15 (2.0)
Carbapenemase producers colonization	19 (2.5)
MRSA colonization	9 (1.2)
*P. aeruginosa* MDR colonization	5 (0.7)
Site of primary infection, *n* (%)	
*Urinary tract*	253 (33.4)
Abdomen	152 (20.1)
Lung	76 (10.0)
Skin and soft tissues	57 (7.5)
Prothesis or implantable device	37 (4.9)
Central nervous system	7 (0.9)
Other sites	30 (4.0)
Unknown origin	159 (21.0)
*Clinical presentation*	
SOFA score, median (IQR)	3 (1–4)
Sepsis, *n* (%)	423 (55.9)
Septic shock, *n* (%)	107 (14.1)

*n* number, *IQR* interquartile range, *EPG* Endoscopic Percutaneous Gastrostomy, *ESBL* extended spectrum beta lactamase, *LTCF* long term care facilities, *MDRO* multi-drug resistant organism, *MRSA* methicillin resistant Staphylococcus aureus, *SOFA* Sequential Organ Failure Assessment

## Results

### Characteristics of participants

**Fig. 1:**
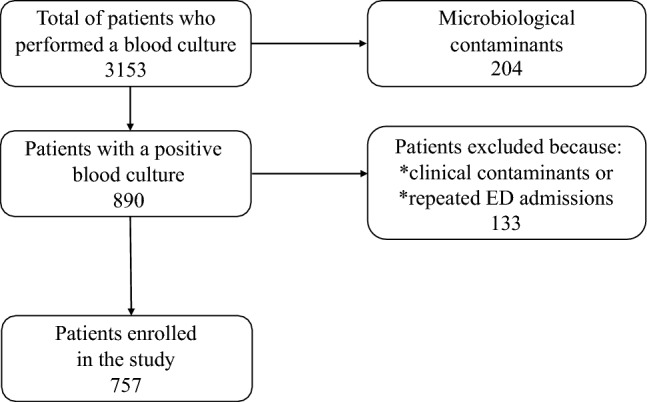
Flow diagram of the study population. *ED* emergency department

During the study period, a blood culture was performed on 3153 patients in the ED, out of which 890 (28%) patients tested positive. Following the exclusion of blood cultures defined as clinical contaminants, as well as those performed for a repeat admission during the study period, 757 consecutive patients with positive blood cultures are included in the analysis, as shown in Fig. 1.

Of these, 292 (38.6%) were females, and the median age was 71 [IQR 59–80] years.

Demographics, comorbidities, risk factors for MDRO, site of infection, and disease severity are summarized in 1. The median Charlson Comorbidity Index was 5 [IQR 3–7]. The urinary tract was the most frequently affected site of infection (33.4%), followed by abdominal (20.1%) and respiratory infections (10%), A primary BSI was found in 159 patients (21%), in which the site of infection could not be determined. The median acute SOFA score was 3 [IQR 1–4], whereas 423 (55.9%) patients had sepsis and 107 (14.1%) had septic shock.

### Prevalence and risk factors for MDRO, ESBL-producing Enterobacterales, MRSA infections

Microbiological findings are summarized in Table 2. Of the 757 patients included in the study, 69 (8.9%) presented with a polymicrobial BSI. Overall, 832 pathogens were isolated and summarized in Table 2. At least one MDRO was isolated in 156 (20.6%, 95%CI 17.9–23.6%) patients with ESBL-producing *Enterobacterales* being the most prevalent isolates (*n* = 106, 14%, 95%CI 11.6–16.5%), including 75 (9%) patients with ESBL-producing *E. coli* and 16 (1.9%) with ESBL-producing *K. pneumoniae.* The second most frequently observed MDRO was MRSA (*n* = 29, 3.8%, 95%CI 2.7–5.4%).

**Table 2:** Microbiological findings

Polymicrobial infections, *n* (%)	67 (8.9)
MDR infections, *n* (%)	156 (18.7)
Total isolated bacteria, *n* (%)	832
*Gram-negative bacteria*, *n* (%)	552 (66.3)
MDRO	120 (14.4)
*Escherichia coli*	336 (40.4)
ESBL producers	75 (9.0)
Carbapenemase producers	1 (0.1)
*Klebsiella pneumoniae*	69 (8.3)
ESBL producers	16 (1.9)
Carbapenemase producers	6 (0.7)
*Proteus mirabilis*	28 (3.4)
ESBL producers	9 (1.1)
*Enterobacter spp.*	22 (2.6)
ESBL producers	6 (0.7)
*Pseudomonas aeruginosa*	47 (5.6)
MDR	7 (0.8)
Others^a^	50 (6.0)
*Gram-positive bacteria, n *(%)	280 (33.7)
MDRO	35 (4.2)
*Staphylococcus aureus*	105 (12.6)
MRSA	29 (3.5)
CoNS^b^	31 (3.7)
*Enterococcus faecalis*	30 (3.6)
*Enterococcus faecium*	15 (1.8)
VRE	4 (0.5)
*Streptococcus pneumoniae*	29 (3.4)
Others^c^	72 (8.7)

n number, ESBL extended spectrum beta lactamase, MDRO multi-drug resistant organism, MRSA methicillin resistant Staphylococcus aureus

^a^*A. hydrophila, B. fragilis, B. thetaiotaomicron, C. fetus, C. freundii, C. gleum, C. koseri, F. gonidiaformans, F. necrophorum, H. influenzae, M. morganii, P. rettgeri, Pantoea spp., Pseudomonas spp., Salmonella spp., S. marcescens, K. oxytoca*

^b^*Coagulase-Negative staphylococci: S. capitis, S. caprae, S. epidermidis, S. haemolyticus, S. hominis, S. schleiferi*

^c^*A. haemolyticum, B. cereus, C. paraputrificum, C. perfrigens, C. sphenoides, E. avium, E. casseliflavus, E. gallinarum, G. morbillorum, G. adjacens, L. monocytogenes, P. micra, P. micros, S. agalactiae, S. anginosus, S. constellatus, S. dysgalactiae, S. equinus, S. gallolyticus, S. mitis, S. oralis, S. pyogenes, S. salivarius, S. gordonii, S. parasanguinis, S. sanguinis*

**Fig. 2:**
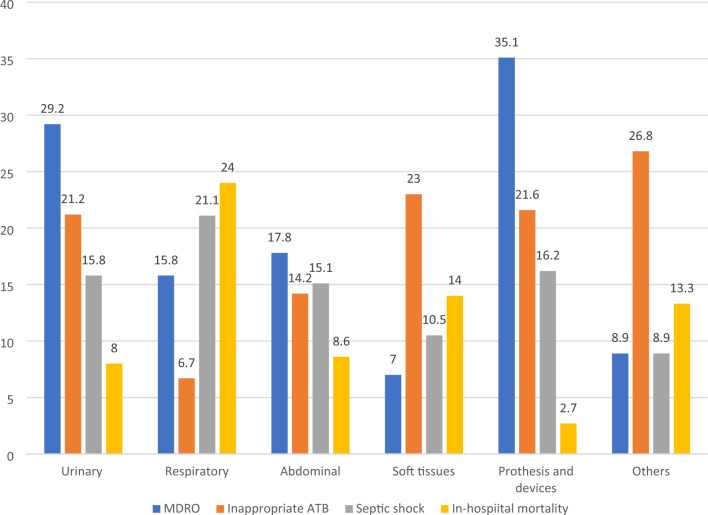
MDRO prevalence, antibiotic inappropriateness, septic shock and in-hospital mortality according to site of infection. Numbers refers to percentages. *MDRO* multi-drug resistant organism, *ATB* antibiotic

Demographics, comorbidities, risk factors, clinical findings, disease severity, and appropriateness of empiric antibiotic therapy according to the presence of MDRO, ESBL-producing *Enterobacterales*, and MRSA are reported in supplemental materials in Tables S1, S2 and S3, respectively. The specific Minimum Inhibiting Concentration (MIC) for piperacillin/ tazobactam, carbapenemic, Ceftolozano/tazobactam and Ceftazidime/avibactam for ESBL-producing *Enterobacterales* is shown in Table S4. Prevalence of MDRO according to the source of infection is reported in Fig. 2. Five hundred and fifty (73%) patients had at least one risk factor for MDRO. The most frequent were antibiotic therapy in the last 90 days (43.1%), hospitalization in the previous three months (40.1%), and day hospital attendance (23.1%). Prevalence of MDRO according to the number of risk factors are reported in Table S5; the presence of at least two risk factors identifies a group of patients with a prevalence equal to or higher than that of the general population.

After adjusting for several confounders, independent risk factors associated with the occurrence of BSI due to MDRO were history of chronic renal failure (OR 2.2; 95% CI 1.4–3.6 *p* < 0.001), nursing home or LTCF residency (OR 4.4; CI 1.9–10.2; *p* < 0.001) and antibiotic therapy in the last 90 days (OR 2.6; 95% CI 1.7–4; *p* < 0.001). Significant independent risk factors for BSI due to ESBL-producing bacteria were chronic renal failure (OR 2.1; 95% CI 1.2–3.7; *p* = 0.006), antibiotic therapy in the last 90 days (OR 2.5; 95% CI 1.5–4.4; *p* < 0.001) and nursing home or LTCF residency (OR 4.9; 95% CI 1.5–12.4; *p* = 0.007). Significant independent risk factors for BSI due to MRSA were dialysis (OR 12.3; 95% CI 1.8–83; *p* = 0.010), antibiotic therapy, hospital admission in the last 90 days (OR 3.6; 95% CI 1.2–10.6; *p* = 0.019) and ureteral stent or nephrostomy (OR 7.8; 95% CI 1.5–40.9; *p* = 0.015). Univariate and multivariate analyses evaluating risk factors for MDRO, ESBL-producing *Enterobacterales* and MRSA infection are reported in Table 3 and Tables S6, S7 and S8.

**Table 3:** Logistic regression analysis to assess the relationship between MDRO, ESBL producer *Enterobacterales* and MRSA infection and demographic and clinical variables

Variables	Univariate analysis		Multivariate analysis	
	OR (95% CI)	*p*-value	OR (95% CI)	*p*-value
*MDRO infection*				
Charlson comorbidity index > 5	1.6 (1.1–2.3)	0.009	1.1 (0.8–1.7)	0.560
Chronic renal failure	2.5 (1.7–3.7)	< 0.001	2.2 (1.4–3.6)	< 0.001
Antibiotic therapy in the last 90 days	3.2 (2.2–4.6)	< 0.001	2.6 (1.7–4)	< 0.001
Hospital admission in the last 90 days	2.3 (1.6–3.3)	< 0.001	1.2 (0.9–1.9)	0.370
Permanent urethral catheter	1.8 (1–3.3)	0.044	1.3 (0.7–2.4)	0.469
Nephrostomy	1.5 (0.6–3.8)	0.435	1.4 (0.7–2.8)	0.343
Dialysis	2.3 (0.9–6)	0.085	2.6 (1–7.1)	0.061
Nursing home or LTCF residency	5.2 (2.4–11.4)	< 0.001	4.4 (1.9–10.2)	< 0.001
*ESBL producer Enterobacteriaceae infection*				
Charlson comorbidity index > 5	1 (1–1.1)	0.147	1.1 (0.7–1.8)	0.713
Chronic renal failure	2.1 (1.3–3.5)	0.002	2.1 (1.2–3.7)	0.006
Dialysis	0.7 (0.1–5.7)	0.704	0.7 (0.1–6.4)	0.757
Antibiotic therapy in the last 90 days	3 (1.9–4.8)	< 0.001	2.5 (1.5–4.4)	0.001
Hospital admission in the last 90 days	2.2 (1.4–3.4)	0.001	1.2 (0.7–2.1)	0.506
Permanent urethral catheter	2 (1–4.2)	0.068	1.5 (0.7–3.4)	0.341
Ureteral stent and/or nephrostomy	1.5 (0.6–3.6)	0.351	1.1 (0.5–2.8)	0.793
Nursing home or LTCF residency	5.1 (1.9–13.8)	0.001	4.9 (1.5–12.4)	0.007
*MRSA infection*				
Chronic renal failure	4.2 (1.6–11)	0.003	3 (1–9.7)	0.058
Dyalisis	5.8 (1–33.4)	0.051	12.3 (1.8– 83)	0.010
Antibiotic therapy in the last 90 days	3.9 (1.6–9.5)	0.003	3.6 (1.2–10.6)	0.019
Hospital admission in the last 90 days	2.6 (1.1–6.2)	0.036		
Ureteral stent and/or nephrostomy	4.9 (1.1–22.2)	0.038	7.8 (1.5–40.9)	0.015
Nursing home or LTCF residency	4.2 (0.7–26.3)	0.130	3.7 (0.5–26.2)	0.189

*ESBL* extended spectrum beta lactamase, *MDRO* multi-drug resistant organism, *MRSA* methicillin resistant *Staphylococcus aureus,* *LTCF* long term care facilities

### Antimicrobial therapy

578 patients (76.4%) were treated with an empirical monotherapy, while the remainder received two or more antibiotics. Based on antimicrobial susceptibility test (AST), treatment was inadequate in 145 (19.2%) of the overall population, 78 (50%) of patients with MDRO, 42 (40%) of patients with ESBL-producing *Enterobacterales*, and 17 (58.6%) of patients with MRSA infection. The appropriateness of antibiotic therapy according to different infection sites is reported in Fig. 2, whereas empirical antibiotic therapy data are reported in Table S9.

### Patients’ outcomes

Forty-five (5.9%) patients were discharged from the ED, while 74 (9.8%) were admitted to the ICU, and the remainder were admitted to general wards or sub-intensive care units. In-hospital mortality was 12.5% (*n* = 94) in the overall population and 26% (*n* = 27) among patients with septic shock. In-hospital mortality rates of those with and without MDRO infection were 13.5% and 12.3%, respectively (*p* = 0.791). Among those with ESBL-producing *Enterobacterales* infection, the mortality rate was 12.4% as compared to 8,7% among those with cephalosporin-sensitive *Enterobacterales* (p = 0.359). In patients with MRSA and MSSA infection, in-hospital mortality rates were 10.3% and 22.2%, respectively (*p* = 0.271).

The median (IQR) LOS was 14 [IQR 9–22] days. A longer LOS was observed in patients with MDRO infection (15 days [IQR 11–25] vs 14 [IQR 9–21]; *p* = 0.007), with ESBL-producing *Enterobacteriaceae* infection (16 days [IQR 11–25] vs 11 [IQR 8–17]; *p* < 0.001), but not in patients with MRSA infection (16 [IQR 11–21] vs 17.5 days [IQR 14–30.3]; *p* = 0.490).

After adjusting for age, comorbidities, microorganism, site of infection, disease severity and antibiotic therapy independent risk factors associated with in-hospital mortality were age (OR 1.4 per 10 years; 95% CI 1.1–1.7; *p* < 0.001), Charlson comorbidity Index > 5 (OR 2.4; 95% CI 1.5–4; *p* < 0.001), lung infection (OR 2.4; 95% CI 1.2–4.8; *p* = 0.013), and septic shock (OR 3.4; 95% CI 1.9–5.9; *p* < 0.001). On the contrary urinary tract infection resulted to be associated to a higher in-hospital survival (OR 0.5; 95% CI 0.3–0.1; *p* = 0.039). Univariate and multivariate analyses evaluating risk factors for mortality are reported in Table 4 and Table S10.

**Table 4:** Logistic regression analysis to assess the relationship between in-hospital mortality and demographic, epidemiological and clinical variables

Variables	Univariate analysis		Multivariate analysis	
	OR (95% CI)	*p*-value	OR (95% CI)	*p*-value
Age, per 10 years	1 (1–1.2)	< 0.001	1.4 (1.1–1.7)	0.001
Charlson comorbidity index > 5	1.2 (1.1–1.3)	< 0.001	2.4 (1.5–4)	0.001
Urinary tract	0.5 (0.3–0.8)	0.009	0.5 (0.3–1)	0.039
Lung	2.5 (1.4–4.5)	0.002	2.4 (1.2–4.8)	0.013
Unknown origin	2 (1.2–3.2)	0.005	1.7 (0.9–3)	0.095
SOFA score, per unit	1.3 (1.2–1.4)	< 0.001		
Septic shock	3 (1.8–5)	< 0.001	3.4 (1.9–5.9)	< 0.001
MDRO infection	1.1 (0.7–1.9)	0.689	1 (0.5–1.9)	0.995
*Enterobacteriaceae* ESBL infection	1.4 (0.8–2.6)	0.244		
MRSA infection	0.4 (0.2–1.5)	0.177		
Appropriate empirical antimicrobial therapy in the emergency room	0.7 (0.4–1.2)	0.223	0.7 (0.4–1.3)	0.311

*ESBL* extended spectrum beta lactamase, *MDRO* multi-drug resistant organism, *MRSA* methicillin resistant Staphylococcus aureus, *SOFA* Sequential Organ Failure Assessment

## Discussion

The study describes the epidemiology of BSI and explores almost all risk factors for MDRO in a large sample of consecutive patients with BSI admitted to the ED during a 3-year period. Some considerations emerge from the results: *first,* almost one in every five patients had an infection due to multi-drug resistant pathogens, with ESBL-producing *Enterobacterales* being the most prevalent ones followed by MRSA. *Second*, nearly three-quarters of patients admitted to the hospital from the community—including those with regular access to healthcare—with a BSI had at least one risk factor for MDRO. However, independent risk factors for infection due to MDRO and ESBL-producing *Enterobacterales* infection were chronic renal failure, nursing home or LTCF residency and antibiotic therapy use in the last 90 days, whereas for MRSA, independent risk factors were dialysis, antibiotic therapy and/or hospital admission in the last 90 days and the presence of ureteral stent or nephrostomy. *Third,* half of the patients with MDRO infection started an empirical antibiotic therapy which is inactive according to the in vitro susceptibility, compared to 11.7% of patients without MDRO infection. *Fourth,* patients with MDRO had a longer LOS but not a higher in-hospital mortality rate. Independent risk factors for in-hospital mortality were higher age, higher Charlson Comorbidity Index, lung infection, and presence of septic shock.

Our study is consistent with previously published data regarding the prevalence of infection sites in patients with BSIs. Urinary tract infections were found to be the most common, followed by abdominal infections. This is likely due to the higher incidence of bloodstream pathogen dissemination from these organ sites [3, 30, 31].

Multidrug-resistant organisms are prevalent (18,7% of the overall population), with ESBL-producing *Enterobacterales* being the most prevalent (13.8%). These results are consistent with the previous observations of a rising incidence of sepsis caused by Gram-negative bacteria, with *E. coli* being the major pathogen among septic patients admitted from the community [1, 4, 32].

We identified a considerably high incidence of risk factors for MDRO. Almost three of every four of our patients exhibited at least one risk factor, a percentage significantly greater than the prevalence of positive culture results for MDRO. A higher number of risk factors identified groups of patients with a higher prevalence of MDRO, suggesting that an appropriate stratification of the risk of MDRO infection weighting different predisposing factors is necessary to choose an appropriate empiric antibiotic therapy. To date, several scores have been published to stratify the risk of MDRO infection more accurately and to help clinicians to decide when to broad antimicrobial spectrum. However, helpful they are, their usefulness in clinical may be limited by different reasons. First, their diagnostic accuracy is highly dependent on the local epidemiology and the characteristics of the population in which they are derived and validated, which makes them sometimes difficult to replicate in other contexts. Secondly, as many are pathogen-specific, they are less easy to use in everyday clinical practice, where the epidemiology is highly variable in a heterogenous population as that observed in the ED. Thirdly, although a more accurate stratification of the risk of MDRO infection is essential for clinicians, defining a risk cut-off for starting a broad-spectrum antibiotic is not obvious and should be tailored considering also other patient’s characteristics (*i.e.* immunodepression), the severity of the infection (*i.e*. presence of sepsis or septic shock), and the possibility to perform rapid microbiological diagnostics (*ie.* molecular biology techniques).

Nursing home or long-term care facility residency was the most significant independent variable among healthcare-related risk factors associated with both MDRO and ESBL-producing *Enterobacterales* BSI, followed by previous antibiotic therapy in the last 90 days and chronic renal failure. All three factors have previously been identified as risk factors and their role in increasing the risk of MDRO infections is understandable [4, 6, 7, 33]. Settings with higher antibiotic selective pressure, with heightened chance of transmission between healthcare workers and patients, together with suboptimal functional state which renders patients vulnerable to frequent hospitalizations and outpatient visits, are pivotal factors in the likelihood of acquiring drug-resistant bacteria [15, 34]. Specific risk factors for BSI due to MRSA were dialysis, ureteral stent or nephrostomy, and previous antibiotic therapy and/or hospitalization within the last 90 days. As known, MRSA is becoming more prevalent in hospital and community settings, and vascular devices, their frequent manipulation, and all others invasive percutaneous or non-percutaneous maneuvers increase the risk of systemic MRSA infections especially when performed in hospital environment [35–38].

Half of patients with infection due to an MDRO received an inappropriate empirical antibiotic therapy compared to 11,7% of patients with a sensitive pathogen. Nonetheless, the outcome aligns with prior research and emphasizes the need for precise risk stratification among patients with MDRO infections to impact on mortality reduction [4]. Currently, this stratification is insufficiently accurate to promptly recognize patients who require a more extensive spectrum antibiotic therapy.

No increase in mortality rate was detected in patients infected with MDRO, despite the higher risk of ineffective empirical therapy. Patients infected with ESBL-producing *Enterobacterales* had a slightly higher mortality rate, though not significant. On the contrary, the multidrug-resistant phenotype has been associated to higher mortality rate in previous studies, given these patients frequently received inactive empirical treatment, resulting in delayed effective therapy [11, 20, 39]. The low prevalence of septic shock in our cohort may in part explain the lack of association of both MDRO phenotype and inactive empirical therapy with outcomes.

Among risk factors for mortality, older age, higher Charlson comorbidity index and the presence of septic shock are easy to understand as they indicate a worse basal status and a more severe infection. In addition, lung infection was also associated with a worse outcome. Pneumonia tends to have a lower rate of positive bacteremia compared to urinary or abdominal infections, and those with hematogenous spread, both community and hospital-acquired, have previously been described as more severe and associated with higher mortality [40, 41]. On the contrary, urinary tract infection was associated with a better prognosis, as widely described in other studies given the higher effectiveness of antimicrobials in this site and the possibility of faster source control.

Inadequate empirical antibiotic therapy was not found to be an independent risk factor for mortality, differently from what previously reported in other cohorts [42, 43] This result must be interpreted with caution, considering the population included and several limitations of the study. First, the effect of inadequate empirical antibiotic therapy on mortality may have been attenuated by the substantial proportion of patients who did not have sepsis or septic shock. Nonetheless, adequacy was defined only by the in vitro susceptibility of the isolated pathogen, whereas the appropriateness according to the site of infection, dosage, route of administration and continuous/intermittent infusion were not assessed, and time-to-adequate antimicrobial therapy was not included in the analysis. Secondly, piperacillin/tazobactam was considered adequate in patients with *Enterobacterales* resistant to third-generation cephalosporins if in vitro susceptibility was reported, although its use in this setting is still controversial [44–46]. Thirdly, no adjustment was made for other therapeutic measures such as fluids, amines, ventilatory support, etc. Fourthly, the effect on mortality was assessed for the whole population and not for specific pathogens or sites of infection, given the small sample size.

A longer LOS was observed in patients with MDRO infection and especially with ESBL-producing *Enterobacterales* infection. The interpretation of these data is certainly limited by the fact that the duration of antibiotic therapy was not recorded; thus, it is difficult to conclude whether a longer LOS was due to a longer duration of therapy rather than other reasons.

In addition, other limitations of the study should be mentioned. The retrospective nature of the study may have limited the quality of data collection, and the small sample size and single-center design may limit the generalizability of the results to other settings. Nonetheless, the single-center design could on the contrary have contributed to the sample homogeneity in terms of supportive measures and diagnostic work up. Moreover, the enrolment period included the COVID-19 pandemic waves, during which a slight reduction and selection of patients admitted to the ED was observed.

## Conclusions

Bloodstream infections due to MDROs are common among patients admitted to the ED, with ESBL-producing *Enterobacterales* and MRSA being the most frequent. As they are often associated with inappropriate empirical antibiotic therapy, a more accurate and early identification of patients at higher risk of MDRO is crucial for the choice of empirical antibiotic therapy. Continuous updating of the local epidemiology and specific risk factors for MDRO help clinicians to select appropriate empirical antibiotic regimens in the ED, while awaiting microbiological results.

## Supplementary Information

Below is the link to the electronic supplementary material.

Supplementary file1 (DOCX 75 KB)

## Data Availability

The datasets used and/or analysed during the current study are available from the corresponding author on reasonable request.

## Abbreviations

**BSI**: Bloodstream infection
**CI**: Confidence interval
**ED**: Emergency department
**ESBL**: Extended-spectrum beta-lactamase
**IQR**: Interquartile range
**LOS**: Length of hospital stay
**LTCF**: Long-term care facility
**MDRO**: Multi-drug resistant organism
**MRSA**: Methicillin-resistant *Staphylococcus aureus*
**SOFA**: Sequential organ failure assessment
