# Supplementary material for: Bloodstream infections due to multi-drug resistant bacteria in the emergency department: prevalence, risk factors and outcomes—a retrospective observational study
Source: Intern Emerg Med. 2024 Jul 13;20(2):573–83. doi: 10.1007/s11739-024-03692-7 (PMC11950129; doi:10.1007/s11739-024-03692-7)
Supplement: Supplementary file 1 — Supplementary file1 (DOCX 75 KB) [file 11739_2024_3692_MOESM1_ESM.docx]

Table S1. Study population according to MDRO infection.

| Variables | Non-MDRO infections | MDRO infections | p-value |
| --- | --- | --- | --- |
| Study population, n (%) | 601 (100.0) | 156 (100.0) |  |
| Demographics characteristics | | | |
| Female | 230 (38.3) | 62 (39.7) | 0.807 |
| Age, years, median (IQR) age | 71 (60-80) | 71 (59-80) | 0.809 |
| Comorbidities, n (%) | | | |
| Charlson comorbidity index, median (IQR) | 5 (3-7) | 5 (4-7) | 0.018 |
| Hypertension | 323 (53.7) | 91 (58.3) | 0.349 |
| Diabetes mellitus | 174 (29.0) | 52 (33.3) | 0.333 |
| Chronic renal failure | 113 (18.8) | 57 (36.5) | <0.001 |
| Solid Cancer | 121 (20.1) | 35 (22.4) | 0.601 |
| Ischemic heart disease | 74 (12.3) | 21 (13.5) | 0.802 |
| Chronic obstructive pulmonary disease | 73 (12.1) | 19 (12.2) | 1.000 |
| Chronic heart failure | 55 (9.2) | 14 (9.0) | 1.000 |
| Hematological malignancy | 50 (8.3) | 15 (9.6) | 0.723 |
| Solid organ transplantation | 45 (7.5) | 16 (10.3) | 0.334 |
| Dementia | 37 (6.2) | 15 (9.6) | 0.179 |
| Chronic liver disease | 36 (6.0) | 13 (8.3) | 0.380 |
| Stroke | 38 (6.3) | 10 (6.4) | 1.000 |
| Bronchiectasis | 13 (2.2) | 3 (1.9) | 1.000 |
| Bone marrow transplantation | 13 (2.2) | 3 (1.9) | 1.000 |
| HIV infection | 10 (1.7) | 4 (2.6) | 0.682 |
| Risk factors for infectious disease and MDRO colonization, n (%) | | | |
| Antibiotic therapy in the last 3 months | 224 (37.3) | 102 (65.4) | <0.001 |
| Hospital admission in the last 3 months | 218 (36.3) | 89 (57.1) | <0.001 |
| Day hospital admission in the last 3 months | 139 (23.1) | 36 (23.1) | 1.000 |
| Chemotherapy in the last 30 days | 86 (14.3) | 15 (9.6) | 0.16 |
| Endovascular devices | 71 (11.8) | 23 (14.7) | 0.394 |
| Immunosuppressive therapy | 55 (9.2) | 19 (12.2) | 0.325 |
| Ulcers and difficult wounds | 49 (8.2) | 12 (7.7) | 0.981 |
| Permanent urethral catheter | 40 (6.7) | 18 (11.5) | 0.061 |
| Biliary stent | 40 (6.7) | 17 (10.9) | 0.105 |
| Chronic corticosteroid therapy | 33 (5.5) | 12 (7.7) | 0.397 |
| Ureteral stent | 18 (3.0) | 11 (7.1) | 0.034 |
| Nephrostomy | 16 (2.7) | 6 (3.8) | 0.605 |
| Dialysis | 12 (2.0) | 7 (4.5) | 0.138 |
| Nursing home or LTCF residency | 12 (2) | 16 (10.2) | 0.001 |
| Intestinal stoma | 10 (1.7) | 2 (1.3) | 1.000 |
| Nasogastric tube or EPG | 10 (1.7) | 2 (1.3) | 1.000 |
| Home parenteral therapy | 5 (0.8) | 2 (1.3) | 0.957 |
| ESBL producers colonization | 9 (1.5) | 6 (3.8) | 0.120 |
| Carbapenemase producers colonization | 9 (1.5) | 10 (6.4) | 0.001 |
| MRSA colonization | 6 (1.0) | 3 (1.9) | 0.593 |
| P. aeruginosa MDRO colonization | 5 (0.8) | 0 (0.0) | 0.556 |
| Site of primary infection, n (%) | | | |
| Urinary tract | 179 (29.8) | 74 (47.4) | <0.001 |
| Abdomen | 126 (21.0) | 26 (16.7) | 0.279 |
| Lung | 64 (10.6) | 12 (7.7) | 0.344 |
| Skin and soft tissues | 53 (8.8) | 4 (2.6) | 0.014 |
| Prothesis or implantable device | 24 (4.0) | 13 (8.3) | 0.042 |
| Central nervous system | 6 (1.0) | 1 (0.6) | 1.000 |
| Other sites | 27 (4.5) | 3 (1.9) | 0.362 |
| Unknown origin | 131 (21.8) | 28 (17.9) | 0.347 |
| Clinical presentation | | | |
| SOFA score, median (IQR) | 3 (2-5) | 4 (2-6) | 0.011 |
| Septic shock, n (%) | 81 (13.5) | 26 (16.7) | 0.374 |
| Outcomes | | | |
| Appropriate empirical antimicrobial therapy in the emergency room | 508 (88.3) | 78 (50.00) | <0.001 |
| Discharged from the emergency department, n (%) | 39 (6.5) | 6 (3.8) | 0.292 |
| Intensive care unit admission, n (%) | 59 (9.8) | 15 (9.6) | 1.000 |
| In-hospital deaths, n (%) | 73 (12.3) | 21 (13.5) | 0.791 |
| Inpatient length of stay, overall, median (IQR) | 13 (8-21) | 15 (10-24) | 0.035 |
| Inpatient length of stay, survived patients, median (IQR) | 14 (9-21) | 15 (11-25) | 0.011 |

n number, IQR interquartile range, EPG Endoscopic Percutaneous Gastrostomy, ESBL extended spectrum beta lactamase, LTCF long term care facilities, MDRO multi-drug resistant organism, MRSA methicillin resistant Staphylococcus aureus, SOFA Sequential Organ Failure Assessment

Table S2. Study population according to Enterobacteriaceae ESBL infection.

| Variables | Non-ESBL producers infections | ESBL producers infections | p-value |
| --- | --- | --- | --- |
| Study population, n (%) | 347 (100.0) | 105 (100.0) |  |
| Demographics characteristics | | | |
| Female, n (%) | 151 (43.5) | 46 (43.8) | 1.000 |
| Median (IQR) age, years | 72 (61-80) | 72 (63-81) | 0.414 |
| Comorbidities, n (%) | | | |
| Charlson comorbidity index, median (IQR) | 5 (3-7) | 5 (4-7) | 0.130 |
| Hypertension | 185 (53.3) | 63 (60.0) | 0.274 |
| Diabetes mellitus | 99 (28.5) | 32 (30.5) | 0.793 |
| Chronic renal failure | 68 (19.6) | 36 (34.3) | 0.003 |
| Solid Cancer | 75 (21.6) | 17 (16.2) | 0.284 |
| Ischemic heart disease | 40 (11.5) | 13 (12.4) | 0.948 |
| Chronic obstructive pulmonary disease | 32 (9.2) | 11 (10.5) | 0.846 |
| Chronic heart failure | 18 (5.2) | 10 (9.5) | 0.166 |
| Hematological malignancy | 25 (7.2) | 9 (8.6) | 0.799 |
| Solid organ transplantation | 35 (10.1) | 12 (11.4) | 0.832 |
| Dementia | 20 (5.8) | 11 (10.5) | 0.146 |
| Chronic liver disease | 20 (5.8) | 9 (8.6) | 0.423 |
| Stroke | 22 (6.3) | 7 (6.7) | 1.000 |
| Bronchiectasis | 8 (2.3) | 3 (2.9) | 1.000 |
| Bone marrow transplantation | 6 (1.7) | 2 (1.9) | 1.000 |
| HIV infection | 2 (0.6) | 3 (2.9) | 0.154 |
| Risk factors for infectious disease and MDRO colonization, n (%) | | | |
| Antibiotic therapy in the last 3 months | 131 (37.8) | 68 (64.8) | <0.001 |
| Hospital admission in the last 3 months | 125 (36.0) | 58 (55.2) | 0.001 |
| Day hospital admission in the last 3 months | 80 (23.1) | 19 (18.1) | 0.346 |
| Chemotherapy in the last 30 days | 49 (14.1) | 7 (6.7) | 0.063 |
| Endovascular devices | 36 (10.4) | 11 (10.5) | 1.000 |
| Immunosuppressive therapy | 38 (11.0) | 13 (12.4) | 0.818 |
| Ulcers and difficult wounds | 18 (5.2) | 8 (7.6) | 0.485 |
| Permanent urethral catheter | 21 (6.1) | 12 (11.4) | 0.101 |
| Biliary stent | 31 (8.9) | 10 (9.5) | 1.000 |
| Chronic corticosteroid therapy | 17 (4.9) | 8 (7.6) | 0.410 |
| Ureteral stent | 12 (3.5) | 7 (6.7) | 0.247 |
| Nephrostomy | 8 (2.3) | 2 (1.9) | 1.000 |
| Dialysis | 5 (1.4) | 1 (1.0) | 1.000 |
| Nursing home or LTCF residency | 7 (2.1) | 10 (9.5) | 0.007 |
| Intestinal stoma | 6 (1.7) | 2 (1.9) | 1.000 |
| Nasogastric tube or EPG | 5 (1.4) | 1 (1.0) | 1.000 |
| Home parenteral therapy | 2 (0.6) | 0 (0.0) | 1.000 |
| ESBL producers colonization | 6 (1.7) | 6 (5.7) | 0.06 |
| Carbapenemase producers colonization | 6 (1.7) | 3 (2.9) | 0.744 |
| MRSA colonization | 3 (0.9) | 1 (1.0) | 1.000 |
| P. aeruginosa MDR colonization | 2 (0.6) | 0 (0.0) | 1.000 |
| Site of primary infection, n (%) | | | |
| Urinary tract | 148 (42.7) | 61 (58.1) | 0.008 |
| Abdomen | 87 (25.1) | 17 (16.2) | 0.078 |
| Lung | 23 (6.6) | 7 (6.7) | 1.000 |
| Skin and soft tissues | 12 (3.5) | 1 (1.0) | 0.311 |
| Prothesis or implantable device | 10 (2.9) | 6 (5.7) | 0.282 |
| Central nervous system | 0 (0.0) | 1 (1.0) | 0.526 |
| Other sites | 7 (2.0) | 1 (1.0) | 0.731 |
| Unknown origin | 69 (19.9) | 15 (14.3) | 0.250 |
| Clinical presentation | | | |
| SOFA score, median (IQR) | 3 (2-5) | 4 (2-6) | 0.438 |
| Septic shock, n (%) | 53 (15.3) | 14 (13.3) | 0.739 |
| Outcomes | | | |
| Appropriate empirical antimicrobial therapy in the emergency room | 312 (92.9) | 63 (60.0) | <0.001 |
| Discharged from the emergency department, n (%) | 24 (6.9) | 5 (4.8) | 0.574 |
| Intensive care unit admission, n (%) | 34 (9.8) | 11 (10.5) | 0.986 |
| In-hospital deaths, n (%) | 30 (8.7) | 13 (12.4) | 0.359 |
| Inpatient length of stay, overall, median (IQR) | 11(7-17) | 15 (10-25) | <0.001 |
| Inpatient length of stay, survived patients, median (IQR) | 11 (8-17) | 16 (11-25) | <0.001 |

n number, IQR interquartile range, EPG Endoscopic Percutaneous Gastrostomy, ESBL extended spectrum beta lactamase, LTCF long term care facilities, MDRO multi-drug resistant organism, MRSA methicillin resistant Staphylococcus aureus, SOFA Sequential Organ Failure Assessment

Table S3. Study population according to MRSA infection.

| Variables | Non-MRSA infections | MRSA infections | p-value |  |
| --- | --- | --- | --- | --- |
| Study population, n (%) | 74 (100.0) | 29 (100.0) |  |  |
| Demographics characteristics | | | | |
| Female, n (%) | 53 (71.6) | 21 (72.4) | 1.000 |  |
| Median (IQR) age, years | 70.5 (58.25, 80.75) | 70.0 (58-78) | 0.439 |  |
| Comorbidities, n (%) | | | | |
| Charlson comorbidity index, median (IQR) | 5 (3-7) | 6 (4-8) | 0.467 |  |
| Hypertension | 43 (58.1) | 13 (48.3) | 0.495 |  |
| Diabetes mellitus | 25 (33.8) | 12 (41.4) | 0.621 |  |
| Chronic renal failure | 12 (16.2) | 13 (44.8) | 0.005 |  |
| Solid Cancer | 16 (21.6) | 9 (31.0) | 0.455 |  |
| Ischemic heart disease | 11 (14.9) | 5 (17.2) | 1.000 |  |
| Chronic obstructive pulmonary disease | 14 (18.9) | 7 (24.1) | 0.749 |  |
| Chronic heart failure | 7 (9.5) | 3 (10.3) | 1.000 |  |
| Hematological malignancy | 4 (5.4) | 3 (10.3) | 0.645 |  |
| Solid organ transplantation | 2 (2.7) | 1 (3.4) | 1.000 |  |
| Dementia | 6 (8.1) | 2 (6.9) | 1.000 |  |
| Chronic liver disease | 6 (8.1) | 1 (3.4) | 0.682 |  |
| Stroke | 7 (9.5) | 2 (6.9) | 0.979 |  |
| Bronchiectasis | 2 (2.7) | 0 (0.0) | 0.920 |  |
| Bone marrow transplantation | 2 (2.7) | 0 (0.0) | 0.920 |  |
| HIV infection | 4 (5.4) | 0 (0.0) | 0.478 |  |
| Risk factors for infectious disease and MDRO colonization, n (%) | | | | |
| Antibiotic therapy in the last 3 months | 22 (29.7) | 18 (62.1) | 0.005 |  |
| Hospital admission in the last 3 months | 24 (32.4) | 16 (55.2) | 0.057 |  |
| Day hospital admission in the last 3 months | 17 (23.0) | 11 (37.9) | 0.198 |  |
| Chemotherapy in the last 30 days | 10 (13.5) | 5 (17.2) | 0.864 |  |
| Endovascular devices | 13 (17.6) | 8 (27.6) | 0.388 |  |
| Immunosuppressive therapy | 2 (2.7) | 1 (3.4) | 1.000 |  |
| Ulcers and difficult wounds | 12 (16.2) | 4 (13.8) | 0.998 |  |
| Permanent urethral catheter | 6 (8.1) | 2 (6.9) | 1.000 |  |
| Biliary stent | 0 (0.0) | 0 (0.0) | - |  |
| Chronic corticosteroid therapy | 4 (5.4) | 2 (6.9) | 1.000 |  |
| Ureteral stent | 1 (1.4) | 3 (10.3) | 0.119 |  |
| Nephrostomy | 2 (2.7) | 4 (13.8) | 0.090 |  |
| Dialysis | 2 (2.7) | 4 (13.8) | 0.090 |  |
| Nursing home or LTCF residency | 2 (2.7) | 3 (10.3) | 0.625 |  |
| Intestinal stoma | 3 (4.1) | 0 (0.0) | 0.653 |  |
| Nasogastric tube or EPG | 0 (0.0) | 1 (3.4) | 0.625 |  |
| Home parenteral therapy | 1 (1.4) | 2 (6.9) | 0.393 |  |
| ESBL producers colonization | 1 (1.4) | 0 (0.0) | 1.000 |  |
| Carbapenemase producers colonization | 0 (0.0) | 1 (3.4) | 0.625 |  |
| MRSA colonization | 1 (1.4) | 2 (6.9) | 0.393 |  |
| P. aeruginosa MDR colonization | 1 (1.4) | 0 (0.0) | 1.000 |  |
| Site of primary infection, n (%) | | | | |
| Urinary tract | 8 (10.8) | 7 (24.1) | 0.157 |  |
| Abdomen | 4 (5.4) | 1 (3.4) | 1.000 |  |
| Lung | 12 (16.2) | 4 (13.8) | 0.998 |  |
| Skin and soft tissues | 12 (16.2) | 3 (10.3) | 0.653 |  |
| Prothesis or implantable device | 11 (14.9) | 7 (24.1) | 0.409 |  |
| Central nervous system | 2 (2.7) | 0 (0.0) | 0.920 |  |
| Other sites | 7 (9.5) | 2 (6.9) | 0.930 |  |
| Unknown origin | 22 (29.7) | 6 (20.7) | 0.496 |  |
| Clinical presentation | | | | |
| SOFA score, median (IQR) | 3 (1-5) | 4 (2-7) | 0.174 |  |
| Septic shock, n (%) | 10 (13.5) | 5 (17.2) | 0.864 |  |
| Outcomes | | | | |
| Appropriate empirical antimicrobial therapy in the emergency room | 68 (94.4) | 12 (41.4) | <0.001 |  |
| Discharged from the emergency department, n (%) | 5 (6.8) | 0 (0.0) | 0.355 |  |
| Intensive care unit admission, n (%) | 9 (12.2) | 3 (10.3) | 1.000 |  |
| In-hospital deaths, n (%) | 16 (22.2) | 3 (10.3) | 0.271 |  |
| Inpatient length of stay, overall, median (IQR) | 16.50 (9.75, 27.25) | 15.50 (10.00, 20.25) | 0.675 |  |
| Inpatient length of stay, survived patients, median (IQR) | 17.50 (14-30.25) | 16 (11-21) | 0.490 |  |

n number, IQR interquartile range, EPG Endoscopic Percutaneous Gastrostomy, ESBL extended spectrum beta lactamase, LTCF long term care facilities, MDRO multi-drug resistant organism, MRSA methicillin resistant Staphylococcus aureus, SOFA Sequential Organ Failure Assessment

Table S4. Minimum Inhibiting Concentration and empiric antibiotic therapy of ESBL-producing Enterobacterales.

| Pathogen | MIC  Piperacillina/  Tazobactam | MIC  Meropenem | MIC  Ceftolozano/ tazobactam | MIC  Ceftazidime/  avibactam | Empirical antibiotic | In vitro susceptibility (1=yes; 0= no) |
| --- | --- | --- | --- | --- | --- | --- |
| Enterobacter aerogenes ESBL | >16 | <=0.12 | - | - | Fluoroquinolone | 1 |
| Enterobacter aerogenes ESBL | >16 | <=0.12 | - | - | Piperacillin/ Tazobactam | 0 |
| Enterobacter cloacae ESBL | <=8 | <=0.12 | - | - | Fluoroquinolone | 1 |
| Enterobacter cloacae ESBL | <=8 | <=0.12 | - | - | ceftriaxone | 0 |
| Enterobacter cloacae ESBL | >16 | <=0.12 | - | - | amoxicillin/clavulanic acid | 0 |
| Enterobacter cloacae ESBL | >16 | <=0.12 | - | - | Carbapenemic | 1 |
| Escherichia coli ESBL | <=4 | <=0.12 | - | - | amoxicillin/clavulanic acid | 0 |
| Escherichia coli ESBL | <=4 | <=0.12 | - | - | ceftazidime | 0 |
| Escherichia coli ESBL | <=4 | <=0.12 | - | - | Carbapenemic | 1 |
| Escherichia coli ESBL | <=4 | <=0.12 | - | - | Piperacillin/ Tazobactam | 1 |
| Escherichia coli ESBL | <=4 | <=0.12 | - | - | Piperacillin/ Tazobactam | 1 |
| Escherichia coli ESBL | <=8 | <=0.12 | - | - | Piperacillin/ Tazobactam | 1 |
| Escherichia coli ESBL | >16 | <=0.12 | - | - | Piperacillin/ Tazobactam | 0 |
| Escherichia coli ESBL | >16 | <=0.12 | - | - | Piperacillin/ Tazobactam | 0 |
| Escherichia coli ESBL | <=4 | <=0.12 | - | - | amoxicillin/clavulanic acid | 1 |
| Escherichia coli ESBL | >16 | <=0.12 | - | - | Fluoroquinolone | 0 |
| Escherichia coli ESBL | <=4 | <=0.12 | - | - | Carbapenemic | 1 |
| Escherichia coli ESBL | >16 | <=0.12 | - | - | Piperacillin/ Tazobactam | 0 |
| Escherichia coli ESBL | <=8 | <=0.12 | - | - | amoxicillin/clavulanic acid | 0 |
| Escherichia coli ESBL | <=4 | <=0.12 | - | - | Piperacillin/ Tazobactam | 1 |
| Escherichia coli ESBL | <=4 | <=0.12 | - | - | Piperacillin/ Tazobactam | 1 |
| Escherichia coli ESBL | >16 | 0.12 | - | - | Piperacillin/ Tazobactam | 0 |
| Escherichia coli ESBL | <=4 | <=0.12 | - | - | Piperacillin/ Tazobactam | 1 |
| Escherichia coli ESBL | <=8 | <=0.12 | - | - | Piperacillin/ Tazobactam | 1 |
| Escherichia coli ESBL | >16 | <=0.12 | - | - | Piperacillin/ Tazobactam | 0 |
| Escherichia coli ESBL | <=4 | <=0.12 | - | - | ceftriaxone | 0 |
| Escherichia coli ESBL | <=4 | <=0.12 | - | - | Carbapenemic | 1 |
| Escherichia coli ESBL | >16 | <=0.12 | - | - | Piperacillin/ Tazobactam | 0 |
| Escherichia coli ESBL | <=4 | <=0.12 | - | - | Carbapenemic | 1 |
| Escherichia coli ESBL | <=8 | <=0.12 | - | - | amoxicillin/clavulanic acid | 0 |
| Escherichia coli ESBL | <=4 | <=0.12 | - | - | Piperacillin/ Tazobactam | 1 |
| Escherichia coli ESBL | <=4 | <=0.12 | - | - | Piperacillin/ Tazobactam | 1 |
| Escherichia coli ESBL | <=4 | <=0.12 | - | - | ceftriaxone | 0 |
| Escherichia coli ESBL | >16 | <=0.12 | - | - | Piperacillin/ Tazobactam | 0 |
| Escherichia coli ESBL | 16 | <=0.12 | - | - | Piperacillin/ Tazobactam | 0 |
| Escherichia coli ESBL | <=4 | <=0.12 | - | - | amoxicillin/clavulanic acid | 0 |
| Escherichia coli ESBL | 16 | <=0.12 | - | - | Carbapenemic | 1 |
| Escherichia coli ESBL | <=4 | <=0.12 | - | - | Piperacillin/ Tazobactam | 1 |
| Escherichia coli ESBL | <=4 | <=0.12 | - | - | Piperacillin/ Tazobactam | 1 |
| Escherichia coli ESBL | <=4 | <=0.12 | - | - | Carbapenemic | 1 |
| Escherichia coli ESBL | <=8 | <=0.12 | - | - | Carbapenemic | 1 |
| Escherichia coli ESBL | 16 | <=0.12 | - | - | ceftriaxone | 0 |
| Escherichia coli ESBL | <=4 | <=0.12 | - | - | ceftriaxone | 0 |
| Escherichia coli ESBL | >16 | <=0.12 | - | - | Piperacillin/ Tazobactam | 0 |
| Escherichia coli ESBL | >16 | <=0.12 | - | - | Carbapenemic | 1 |
| Escherichia coli ESBL | <=4 | <=0.12 | - | - | Carbapenemic | 1 |
| Escherichia coli ESBL | <=8 | <=0.12 | - | - | Piperacillin/ Tazobactam | 1 |
| Escherichia coli ESBL | <=4 | <=0.12 | 0.5/4 | <1/4 | Fluoroquinolone | 1 |
| Escherichia coli ESBL | <=8 | <=0.12 | - | - | Piperacillin/ Tazobactam | 1 |
| Escherichia coli ESBL | 16 | <=0.12 | - | - | Piperacillin/ Tazobactam | 0 |
| Escherichia coli ESBL | <=4 | <=0.12 | - | - | Piperacillin/ Tazobactam | 1 |
| Escherichia coli ESBL | <=4 | <=0.12 | - | - | Fluoroquinolone | 0 |
| Escherichia coli ESBL | 16 | <=0.12 | - | - | ceftriaxone | 0 |
| Escherichia coli ESBL | <=4 | <=0.12 | - | - | Piperacillin/ Tazobactam | 1 |
| Escherichia coli ESBL | <=4 | <=0.12 | - | - | Carbapenemic | 1 |
| Escherichia coli ESBL | <=4 | <=0.12 | - | - | Piperacillin/ Tazobactam | 1 |
| Escherichia coli ESBL | <=4 | <=0.12 | - | - | amoxicillin/clavulanic acid | 0 |
| Escherichia coli ESBL | <=4 | <=0.12 | - | - | Piperacillin/ Tazobactam | 1 |
| Escherichia coli ESBL | <=4 | <=0.12 | - | - | Piperacillin/ Tazobactam | 1 |
| Escherichia coli ESBL | >16 | <=0.12 | - | - | Piperacillin/ Tazobactam | 0 |
| Escherichia coli ESBL | <=4 | <=0.12 | - | - | ceftriaxone | 0 |
| Escherichia coli ESBL | <=4 | <=0.12 | <0.2/4 | <1/4 | ceftriaxone | 0 |
| Escherichia coli ESBL | <=4 | <=0.12 | - | - | Piperacillin/ Tazobactam | 1 |
| Escherichia coli ESBL | <=4 | <=0.12 | - | - | Piperacillin/ Tazobactam | 1 |
| Escherichia coli ESBL | >16 | <=0.12 | - | - | Piperacillin/ Tazobactam | 0 |
| Escherichia coli ESBL | >16 | <=0.12 | - | - | Carbapenemic | 1 |
| Escherichia coli ESBL | <=4 | <=0.12 | - | - | Piperacillin/ Tazobactam | 1 |
| Escherichia coli ESBL | <=8 | <=0.12 | - | - | ceftriaxone | 0 |
| Escherichia coli ESBL | <=8 | <=0.12 | - | - | Piperacillin/ Tazobactam | 0 |
| Escherichia coli ESBL | >16 | <=0.12 | - | - | Piperacillin/ Tazobactam | 0 |
| Escherichia coli ESBL | <=8 | <=0.12 | - | - | Carbapenemic | 1 |
| Escherichia coli ESBL | <=4 | <=0.12 | - | - | amoxicillin/clavulanic acid | 1 |
| Escherichia coli ESBL | <=4 | <=0.12 | - | - | Piperacillin/ Tazobactam | 1 |
| Escherichia coli ESBL | 16 | <=0.12 | - | - | Piperacillin/ Tazobactam | 0 |
| Escherichia coli ESBL | <=4 | <=0.12 | - | - | ceftriaxone | 0 |
| Escherichia coli ESBL | <=4 | <=0.12 | - | - | Piperacillin/ Tazobactam | 1 |
| Escherichia coli ESBL | <=8 | <=0.12 | - | - | Piperacillin/ Tazobactam | 1 |
| Escherichia coli ESBL | <=4 | <=0.12 | - | - | amoxicillin/clavulanic acid | 1 |
| Escherichia coli ESBL | <=4 | <=0.12 | - | - | Piperacillin/ Tazobactam | 1 |
| Escherichia coli ESBL | >16 | <=0.12 | <=2 | <=1 | Piperacillin/ Tazobactam | 0 |
| Escherichia coli ESBL | <=8 | <=0.12 | <=2 | <=1 | amoxicillin/clavulanic acid | 1 |
| Klebsiella pneumoniae ESBL | >16 | <=0.12 | - | - | Piperacillin/ Tazobactam | 0 |
| Klebsiella pneumoniae ESBL | <=4 | <=0.12 | - | - | Piperacillin/ Tazobactam | 1 |
| Klebsiella pneumoniae ESBL | >16 | <=0.12 | - | - | Piperacillin/ Tazobactam | 0 |
| Klebsiella pneumoniae ESBL | <4 | 4.0 | - | - | amoxicillin/clavulanic acid | 1 |
| Klebsiella pneumoniae ESBL | >16 | <=0.12 | - | - | Piperacillin/ Tazobactam | 0 |
| Klebsiella pneumoniae ESBL | >16 | <=0.12 | - | - | Carbapenemic | 1 |
| Klebsiella pneumoniae ESBL | <=4 | <=0.12 | - | - | Piperacillin/ Tazobactam | 1 |
| Klebsiella pneumoniae ESBL | <=4 | <=0.12 | - | - | Piperacillin/ Tazobactam | 1 |
| Klebsiella pneumoniae ESBL | >16 | <=0.12 | - | - | Piperacillin/ Tazobactam | 0 |
| Klebsiella pneumoniae ESBL | <=8 | <=0.12 | - | - | Piperacillin/ Tazobactam | 1 |
| Klebsiella pneumoniae ESBL | <=4 | <=0-12 | - | - | Carbapenemic | 1 |
| Klebsiella pneumoniae ESBL | <=4 | <=0.12 | - | - | Piperacillin/ Tazobactam | 1 |
| Klebsiella pneumoniae ESBL | <=4 | <=0.12 | - | - | Piperacillin/ Tazobactam | 1 |
| Klebsiella pneumoniae ESBL | <=8 | <=0.12 | - | - | Carbapenemic | 1 |
| Klebsiella pneumoniae ESBL | <=4 | <=0.12 | - | - | Piperacillin/ Tazobactam | 1 |
| Klebsiella pneumoniae ESBL | 16 | <=0.12 | <=1 | <=2 | Carbapenemic | 1 |
| Proteus mirabilis ESBL | >16 | 1 | - | - | Piperacillin/ Tazobactam | 0 |
| Proteus mirabilis ESBL | <=4 | <=0.12 | - | - | Piperacillin/ Tazobactam | 1 |
| Proteus mirabilis ESBL | >16 | <=0.12 | - | - | Carbapenemic | 1 |
| Proteus mirabilis ESBL | <=4 | <=0.12 | - | - | Carbapenemic | 1 |
| Proteus mirabilis ESBL | <=4 | <=0.12 | - | - | Piperacillin/ Tazobactam | 1 |
| Proteus mirabilis ESBL | <=4 | <=0.12 | - | - | Piperacillin/ Tazobactam | 0 |
| Proteus mirabilis ESBL | <=4 | <=0.12 | - | - | Piperacillin/ Tazobactam | 1 |
| Proteus mirabilis ESBL | <=4 | <=0.12 | - | - | Carbapenemic | 1 |
| Proteus mirabilis ESBL | <=4 | <=0.12 | - | - | Piperacillin/ Tazobactam | 1 |

Table S5. Prevalence of MDRO pathogens according to number of risk factors.

| Risk Factors  N° | Non-MDRO  Infections,  n (%) | MDRO  Infections,  n (%) |
| --- | --- | --- |
| 0 | 157 (91.9) | 15 (8.7) |
| 1 | 134 (85.3) | 23 (14.7) |
| 2 | 101 (78.9) | 28 (21) |
| 3 | 81 (74.3) | 28 (25.7) |
| 4 | 71 (70.3) | 30 (29.7) |
| 5 | 35 (62.5) | 21 (37.5) |
| 6 | 13 (61.9) | 8 (38.1) |
| 7 | 8 (80) | 2 (20) |
| 8 | 1 (50) | 1 (50) |
| 9 | 0 | 1 (100) |
| Totale | 601 (79.4) | 156 (20.5) |

n number, MDRO multi-drug resistant organism

Table S6. Logistic regression analysis to assess the relationship between MDRO infection and demographic, epidemiological, clinical variables.

| Variables | Univariate analysis | | Multivariate analysis | | |
| --- | --- | --- | --- | --- | --- |
|  | OR (95% CI) | p-value | OR (95% CI) | p-value |  |
| Demographics characteristics | | | | | |
| Sex F | 1.1 (0.7-1.5) | 0.736 |  |  |  |
| Age, per 10 years | 1 (0.9-1.2) | 0.598 |  |  |  |
| Comorbidities | | | | | |
| Charlson comorbidity index >5 | 1.6 (1.1-2.3) | 0.009 | 1.1 (0.8-1.7) | 0.560 |  |
| Hypertension | 1.2 (0.8-1.7) | 0.305 |  |  |  |
| Diabetes mellitus | 1.2 (0.8-1.8) | 0.287 |  |  |  |
| Chronic renal failure | 2.5 (1.7-3.7) | <0.001 | 2.2 (1.4-3.6) | <0.001 |  |
| Dyalisis | 2.3 (0.9-6) | 0.085 |  |  |  |
| Solid Cancer | 1.1 (0.8-1.8) | 0.527 |  |  |  |
| Ischemic heart disease | 1.1 (0.7-1.9) | 0.700 |  |  |  |
| Chronic obstructive pulmonary disease | 1 (0.6-1.7) | 0.991 |  |  |  |
| Chronic heart failure | 1 (0.5-1.8) | 0.945 |  |  |  |
| Hematological malignancy | 1.2 (0.6-2.1) | 0.607 |  |  |  |
| Solid organ transplantation | 1.4 (0.8-2.6) | 0.260 |  |  |  |
| Dementia | 1.6 (0.9-3) | 0.131 |  |  |  |
| Chronic liver disease | 1.4 (0.7-2.8) | 0.291 |  |  |  |
| Stroke | 1 (0.5-2.1) | 0.968 |  |  |  |
| Bronchiectasis | 0.9 (0.3-3.2) | 0.853 |  |  |  |
| Bone marrow transplantation | 0.9 (0.3-3.2) | 0.853 |  |  |  |
| HIV infection | 1.6 (0.5-5) | 0.461 |  |  |  |
| Risk factors for MDRO infection | | | | | |
| Antibiotic therapy in the last 90 days | 3.2 (2.2-4.6) | <0.001 | 2.6 (1.7-4) | <0.001 |  |
| Hospital admission in the last 90 days | 2.3 (1.6-3.3) | <0.001 | 1.2 (0.9-1.9) | 0.370 |  |
| Day hospital admission in the last 90 days | 1 (0.7-1.5) | 0.989 |  |  |  |
| Chemotherapy in the last 30 days | 0.6 (0.4-1.1) | 0.127 |  |  |  |
| Endovascular devices | 1.3 (0.8-2.1) | 0.324 |  |  |  |
| Immunosuppressive therapy | 1.4 (0.8-2.4) | 0.258 |  |  |  |
| Ulcers and difficult wounds | 1 (0.5-1.8) | 0.851 |  |  |  |
| Permanent urethral catheter | 1.8 (1-3.3) | 0.044 | 1.3 (0.7-2.4) | 0.469 |  |
| Biliary stent | 1.7 (0.9-3.1) | 0.077 |  |  |  |
| Chronic corticosteroid therapy | 1.4 (0.7-2.8) | 0.302 |  |  |  |
| Ureteral stent | 2.5 (1.1-5.3) | 0.022 |  |  |  |
| Nephrostomy | 1.5 (0.6-3.8) | 0.435 | 1.4 (0.7-2.8) | 0.343 |  |
| Nursing home or LTCF residency | 5.2 (2.4-11.4) | <0.001 | 4.4 (1.9-10.2) | <0.001 |  |
| Intestinal stoma | 0.7 (0.2-3.5) | 0.734 |  |  |  |
| Nasogastric tube or EPG | 0.8 (0.2-3.5) | 0.734 |  |  |  |
| Home parenteral therapy | 1.5 (0.3-8.1) | 0.604 |  |  |  |
| ESBL producers colonization | 2.6 (0.9-7.5) | 0.071 |  |  |  |
| Carbapenemase producers colonization | 4.5 (1.8-11.3) | 0.001 |  |  |  |
| MRSA colonization | 1.9 (0.5-7.8) | 0.351 |  |  |  |
| Site of primary infection | | | | | |
| Urinary tract | 2.1 (1.5-3) | <0.001 |  |  |  |
| Abdomen | 0.8 (0.5-1.2) | 0.234 |  |  |  |
| Lung | 0.7 (0.4-1.3) | 0.276 |  |  |  |
| Skin and soft tissues | 0.3 (0.1-0.8) | 0.013 |  |  |  |
| Prothesis or implantable device | 2.2 (1.1-4.4) | 0.028 |  |  |  |
| Central nervous system | 0.6 (0.2-5.4) | 0.680 |  |  |  |
| Other sites | 0.4 (0.1-1.4) | 0.149 |  |  |  |
| Unknown origin | 0.8 (0.5-1.2) | 0.294 |  |  |  |
| Clinical presentation | | | | | |
| SOFA score, per unit | 1.1 (1-1.1) | 0.014 |  |  |  |

EPG Endoscopic Percutaneous Gastrostomy, ESBL extended spectrum beta lactamase, LTCF long term care facilities, MDRO multi-drug resistant organism, MRSA methicillin resistant Staphylococcus aureus, SOFA Sequential Organ Failure Assessment

Table S7. Logistic regression analysis to assess the relationship between ESBL producers *Enterobacteriaceae* infection and demographic, epidemiological, clinical variables.

| Variables | Univariate analysis | | Multivariate analysis | | |
| --- | --- | --- | --- | --- | --- |
|  | OR (95% CI) | p-value | OR (95% CI) | p-value |  |
| Demographics characteristics | | | | | |
| Sex F | 1.0 (0.7-1.6) | 0.958 |  |  |  |
| Age, per 10 years | 1.008 (0.9-1.1) | 0.301 |  |  |  |
| Comorbidities | | | | | |
| Charlson comorbidity index >5 | 1 (1-1.1) | 0.147 | 1.1 (0.7-1.8) | 0.713 |  |
| Hypertension | 1.3 (0.8-2) | 0.228 |  |  |  |
| Diabetes mellitus | 1.1 (0.7-1.8) | 0.700 |  |  |  |
| Chronic renal failure | 2.1 (1.3-3.5) | 0.002 | 2.1 (1.2-3.7) | 0.006 |  |
| Dyalisis | 0.7 (0.1-5.7) | 0.704 |  |  |  |
| Solid Cancer | 0.7 (0.4-1.3) | 0.228 |  |  |  |
| Ischemic heart disease | 1.1 (0.6-2.1) | 0.812 |  |  |  |
| Chronic obstructive pulmonary disease | 1.2 (0.6-2.4) | 0.701 |  |  |  |
| Chronic heart failure | 1.9 (0.9-4.3) | 0.112 |  |  |  |
| Hematological malignancy | 1.2 (0.5-2.7) | 0.642 |  |  |  |
| Solid organ transplantation | 1.2 (0.6-2.3) | 0.693 |  |  |  |
| Dementia | 1.9 (0.9-4.1) | 0.099 |  |  |  |
| Chronic liver disease | 1.5 (0.7-3.5) | 0.307 |  |  |  |
| Stroke | 1.1 (0.4-2.5) | 0.905 |  |  |  |
| Bronchiectasis | 1.2 (0.3-4.8) | 0.748 |  |  |  |
| Bone marrow transplantation | 1.1 (0.2-5.6) | 0.905 |  |  |  |
| HIV infection | 5.1 (0.8-30.8) | 0.077 |  |  |  |
| Risk factors for MDRO infection | | | | | |
| Antibiotic therapy in the last 90 days | 3 (1.9-4.8) | <0.001 | 2.5 (1.5-4.4) | 0.001 |  |
| Hospital admission in the last 90 days | 2.2 (1.4-3.4) | 0.001 | 1.2 (0.7-2.1) | 0.506 |  |
| Day hospital admission in the last 90 days | 0.7 (0.4-1.3) | 0.283 |  |  |  |
| Chemotherapy in the last 30 days | 0.4 (0.2-1) | 0.047 |  |  |  |
| Endovascular devices | 1 (0.5-2.1) | 0.976 |  |  |  |
| Immunosuppressive therapy | 1.1 (0.6-2.2) | 0.685 |  |  |  |
| Ulcers and difficult wounds | 1.5 (0.6-3.6) | 0.351 |  |  |  |
| Permanent urethral catheter | 2 (1-4.2) | 0.068 | 1.5 (0.7-3.4) | 0.341 |  |
| Biliary stent | 1.1 (0.5-2.3) | 0.854 |  |  |  |
| Chronic corticosteroid therapy | 1.6 (0.7-3.8) | 0.289 |  |  |  |
| Ureteral stent | 2 (0.8-5.2) | 0.158 |  |  |  |
| Nephrostomy | 0.8 (0.2-3.9) | 0.807 |  |  |  |
| Ureteral stent and/or nephrostomy | 1.5 (0.6-3.6) | 0.351 | 1.1 (0.5-2.8) | 0.793 |  |
| Nursing home or LTCF residency | 5.1 (1.9-13.8) | 0.001 | 4.9 (1.5-12.4) | 0.007 |  |
| Intestinal stoma | 1.1 (0.2-5.6) | 0.905 |  |  |  |
| Nasogastric tube or EPG | 0.7 (0.1-5.7) | 0.704 |  |  |  |
| ESBL producers colonization | 3.4 (1.1-10.9) | 0.036 |  |  |  |
| Carbapenemase producers colonization | 1.7 (0.4-6.8) | 0.473 |  |  |  |
| MRSA colonization | 1.1 (0.1-10.7) | 0.933 |  |  |  |
| Site of primary infection | | | | | |
| Urinary tract | 1.9 (1.2-2.9) | 0.006 |  |  |  |
| Abdomen | 0.6 (0.3-1) | 0.060 |  |  |  |
| Lung | 1 (0.4-2.4) | 0.989 |  |  |  |
| Skin and soft tissues | 0.3 (0-2.1) | 0.209 |  |  |  |
| Prothesis or implantable device | 2 (0.7-5.8) | 0.177 |  |  |  |
| Central nervous system | - | - |  |  |  |
| Other sites | 0.5 (0.1-4.6) | 0.576 |  |  |  |
| Unknown origin | 0.7 (0.4-1.2) | 0.198 |  |  |  |
| Clinical presentation | | | | | |
| SOFA score, per unit | 1 (0.9-1.1) | 0.481 |  |  |  |

EPG Endoscopic Percutaneous Gastrostomy, ESBL extended spectrum beta lactamase, LTCF long term care facilities, MDRO multi-drug resistant organism, MRSA methicillin resistant Staphylococcus aureus, SOFA Sequential Organ Failure Assessment

Table S8. Logistic regression analysis to assess the relationship between MRSA infection and demographic, epidemiological, clinical variables.

| Variables | Univariate analysis | | Multivariate analysis | |
| --- | --- | --- | --- | --- |
|  | OR (95% CI) | p-value | OR (95% CI) | p-value |
| Demographics characteristics | | | | |
| Sex F | 1 (0.4-2.5) | 0.936 |  |  |
| Age, per 10 years | 1 (0.9-1) | 0.268 |  |  |
| Comorbidities | | | | |
| Charlson comorbidity index >5 | 1.1 (0.9-1.2) | 0.489 |  |  |
| Hypertension | 0.7 (0.3-1.6) | 0.368 |  |  |
| Diabetes mellitus | 1.4 (0.6-3.3) | 0.471 |  |  |
| Chronic renal failure | 4.2 (1.6-11) | 0.003 | 3 (1-9.7) | 0.058 |
| Dyalisis | 5.8 (1-33.4) | 0.051 | 12.3 (1.8- 83) | 0.010 |
| Solid Cancer | 1.6 (0.6-4.3) | 0.319 |  |  |
| Ischemic heart disease | 1.2 (0.4-3.8) | 0.765 |  |  |
| Chronic obstructive pulmonary disease | 1.4 (0.5-3.8) | 0.555 |  |  |
| Chronic heart failure | 1.1 (0.3-4.6) | 0.891 |  |  |
| Hematological malignancy | 2 (0.4-9.6) | 0.378 |  |  |
| Solid organ transplantation | 1.3 (0.1-14.7) | 0.840 |  |  |
| Dementia | 0.8 (0.2-4.4) | 0.836 |  |  |
| Chronic liver disease | 0.4 (0-3.5) | 0.412 |  |  |
| Stroke | 0.7 (0.1-3.6) | 0.680 |  |  |
| Risk factors for MDRO infection | | | | |
| Antibiotic therapy in the last 90 days | 3.9 (1.6-9.5) | 0.003 | 3.6 (1.2-10.6) | 0.019 |
| Hospital admission in the last 90 days | 2.6 (1.1-6.2) | 0.036 |  |  |
| Day-hospital admission  in the last 90 days | 2 (0.8-5.2) | 0.129 |  |  |
| Chemotherapy in the last 30 days | 1.3 (0.4-4.3) | 0.630 |  |  |
| Endovascular devices | 1.8 (0.7-4.9) | 0.260 |  |  |
| Immunosuppressive therapy | 1.3 (0.1-14.7) | 0.840 |  |  |
| Ulcers and difficult wounds | 0.8 (0.2-2.8) | 0.760 |  |  |
| Permanent urethral catheter | 0.8 (0.2-4.4) | 0.836 |  |  |
| Chronic corticosteroid therapy | 1.3 (0.2-7.5) | 0.772 |  |  |
| Ureteral stent | 8.4 (0.8-84.6) | 0.070 |  |  |
| Nephrostomy | 5.8 (1-33.4) | 0.051 |  |  |
| Ureteral stent and/or nephrostomy | 4.9 (1.1-22.2) | 0.038 | 7.8 (1.5-40.9) | 0.015 |
| Dialysis | 5.8 (1-33.4) | 0.051 | 12.259 (1.8-83) | 0.010 |
| Nursing home or LTCF residency | 4.2(0.7-26.3) | 0.130 | 3.7 (0.5-26.2) | 0.189 |
| Home parenteral therapy | 5.4 (0.5-62.1) | 0.175 |  |  |
| MRSA colonization | 5.4 (0.5-62.1) | 0.175 |  |  |
| Site of primary infection | | | | |
| Urinary tract | 2.62(0.9-8.1) | 0.092 |  |  |
| Abdomen | 0.6 (0.1-5.8) | 0.680 |  |  |
| Lung | 0.8 (0.2-2.8) | 0.760 |  |  |
| Skin and soft tissues | 0.6 (0.2-2.3) | 0.451 |  |  |
| Prothesis or implantable device | 1.8 (0.6-5.3) | 0.269 |  |  |
| Central nervous system | - | - |  |  |
| Other sites | 0.7 (0.1-3.6) | 0.680 |  |  |
| Unknown origin | 0.6 (0.2-1.7) | 0.356 |  |  |
| Clinical presentation | | | | |
| SOFA score, per unit | 1.1 (1-1.3) | 0.161 |  |  |

LTCF long term care facilities, MDRO multi-drug resistant organism, MRSA methicillin resistant Staphylococcus aureus, SOFA Sequential Organ Failure Assessment

Table S9. Empirical antibiotic therapy started in the Emergency Department and appropriateness according to in vitro susceptibility.

| Antimicrobial agent | Patients,  n (%) | Inappropriate according to in vitro susceptibility,  n (%)* |
| --- | --- | --- |
| Total | 757 (100) | 145 (19.6) |
| No antimicrobial therapy in the emergency room | 29 (3.8) | / |
| Amikacin | 4 (0.5) | 0 (0.0) |
| Amoxicillin Clavulanate | 79 (10.4) | 21 (26.5) |
| Ampicillin | 7 (0.9) | 3 (42.9) |
| Azithromycin | 1 (0.1) | 0 (0.0) |
| Trimethoprim Sulfamethoxazole | 2 (0.3) | 0 (0.0) |
| Ceftazidime | 9 (1.2) | 2 (22.2) |
| Ceftriaxone | 152 (20.1) | 35 (23.0) |
| Ciprofloxacin | 15 (2.0) | 8 (53.3) |
| Clarithromycin | 21 (2.8) | 4 (19.0) |
| Clindamycin | 6 (0.8) | 1 (16.7) |
| Daptomycin | 17 (2.2) | 1 (5.9) |
| Doxycycline | 1 (0.1) | 0 (0.0) |
| Gentamycin | 6 (0.8) | 3 (50.0) |
| Levofloxacin | 61 (8.1) | 6 (9.8) |
| Linezolid | 16 (2.1) | 1 (6.2) |
| Meropenem | 67 (8.9) | 12 (17.9) |
| Metronidazole | 11 (1.5) | 1 (9.1) |
| Moxifloxacin | 1 (0.1) | 1 (100.0) |
| Oxacillin | 1 (0.1) | 0 (0.0) |
| Piperacillin Tazobactam | 356 (47.0) | 55 (15.4) |
| Tigecycline | 3 (0.4) | 0 (0.0) |
| Vancomycin | 45 (5.9) | 0 (0.0) |

* Percentages refer to the total number of patients receiving every single antibiotic.

Table S10. Logistic regression analysis to assess the relationship between in-hospital mortality and demographic, epidemiological and clinical variables.

| Variables | Univariate analysis | | Multivariate analysis | | |
| --- | --- | --- | --- | --- | --- |
|  | OR (95% CI) | p-value | OR (95% CI) | p-value |  |
| Demographics characteristics | | | | | |
| Sex F | 1.3 (0.8-2) | 0.262 |  |  |  |
| Age, per 10 years | 1 (1-1.2) | <0.001 | 1.4 (1.1-1.7) | 0.001 |  |
| Comorbidities | | | | | |
| Charlson comorbidity index >5 | 1.2 (1.1-1.3) | <0.001 | 2.4 (1.5-4) | 0.001 |  |
| Hypertension | 1 (0.6-1.5) | 0.922 |  |  |  |
| Diabetes mellitus | 1.2 (0.7-1.8) | 0.515 |  |  |  |
| Chronic renal failure | 1.4 (0.8-2.2) | 0.215 |  |  |  |
| Solid Cancer | 1.7 (1-2.7) | 0.040 |  |  |  |
| Ischemic heart disease | 1.5 (0.8-2.7) | 0.175 |  |  |  |
| Chronic obstructive pulmonary disease | 2.2 (1.3-3.9) | 0.004 |  |  |  |
| Chronic heart failure | 1.4 (0.7-2.7) | 0.369 |  |  |  |
| Hematological malignancy | 1.9 (1-3.6) | 0.060 |  |  |  |
| Solid organ transplantation | 0.5 (0.2-1.3) | 0.151 |  |  |  |
| Dementia | 2 (1-4) | 0.055 |  |  |  |
| Chronic liver disease | 1.7 (0.8-3.6) | 0.182 |  |  |  |
| Stroke | 1.2 (0.5-2.9) | 0.612 |  |  |  |
| Bronchiectasis | 2.4 (0.8-7.6) | 0.139 |  |  |  |
| Bone marrow transplantation | 1.6 (0.5-5.8) | 0.451 |  |  |  |
| HIV infection | 1.2 (0.3-5.3) | 0.840 |  |  |  |
| Risk factors for MDRO infection | | | | | |
| Antibiotic therapy in the last 3 months | 0.8 (0.5-1.2) | 0.325 |  |  |  |
| Hospital admission in the last 3 months | 0.9 (0.6-1.5) | 0.770 |  |  |  |
| Day hospital admission in the last 3 months | 1 (0.6-1.7) | 0.980 |  |  |  |
| Chemotherapy in the last 30 days | 1.6 (0.9-2.9) | 0.086 |  |  |  |
| Endovascular devices | 0.7 (0.3-1.5) | 0.359 |  |  |  |
| Immunosuppressive therapy | 0.8 (0.4-1.8) | 0.641 |  |  |  |
| Ulcers and difficult wounds | 2.5 (1.3-4.7) | 0.004 |  |  |  |
| Permanent urethral catheter | 1.3 (0.6-2.8) | 0.439 |  |  |  |
| Biliary stent | 0.4 (0.1-1.2) | 0.098 |  |  |  |
| Chronic corticosteroid therapy | 1.6 (0.7-3.5) | 0.275 |  |  |  |
| Ureteral stent | 0.5 (0.1-2.2) | 0.360 |  |  |  |
| Nephrostomy | 1.1 (0.3-3.8) | 0.872 |  |  |  |
| Dialysis | 0.8 (0.2-3.6) | 0.791 |  |  |  |
| Nursing home or LTCF residency | 2.1 (0.6-7.9) | 0.257 |  |  |  |
| Intestinal stoma | 0.7 (0.1-5.5) | 0.731 |  |  |  |
| Nasogastric tube or EPG | 4.1 (1.2 -14.4) | 0.026 |  |  |  |
| Home parenteral therapy | 1.4 (0.2-12.1) | 0.759 |  |  |  |
| ESBL producers colonization | 1.1 (0.2-4.9) | 0.923 |  |  |  |
| Carbapenemase producers colonization | 1.9 (0.6-5.9) | 0.263 |  |  |  |
| MRSA colonization | 2 (0.4-9.9) | 0.386 |  |  |  |
| P. aeruginosa MDR colonization | 1.8 (0.2-15.9) | 0.616 |  |  |  |
| Site of primary infection | | | | | |
| Urinary tract | 0.5 (0.3-0.8) | 0.009 | 0.5 (0.3-1) | 0.039 |  |
| Abdomen | 0.6 (0.3-1.1) | 0.101 |  |  |  |
| Lung | 2.5 (1.4-4.5) | 0.002 | 2.4 (1.2-4.8) | 0.013 |  |
| Skin and soft tissues | 1.2 (0.5-2.5) | 0.719 |  |  |  |
| Prothesis or implantable device | 0.2 (0-1.4) | 0.099 |  |  |  |
| Central nervous system | 1.2 (0.1-9.8) | 0.887 |  |  |  |
| Other sites | 1.4 (0.5-3.8) | 0.501 |  |  |  |
| Unknown origin | 2 (1.2-3.2) | 0.005 | 1.7 (0.9-3) | 0.095 |  |
| Clinical presentation | | | | | |
| SOFA score, per unit | 1.3 (1.2-1.4) | <0.001 |  |  |  |
| Septic shock | 3 (1.8-5) | <0.001 | 3.4 (1.9-5.9) | <0.001 |  |
| Microbiological findings |  |  |  |  |  |
| MDRO infection | 1.1 (0.7-1.9) | 0.689 | 1 (0.5-1.9) | 0.995 |  |
| Enterobacteriaceae ESBL infection | 1.4 (0.8-2.6) | 0.244 |  |  |  |
| MRSA infection | 0.4 (0.2-1.5) | 0.177 |  |  |  |
| Outcomes | | | | | |
| Appropriate empirical antimicrobial therapy in the emergency room | 0.7 (0.4-1.2) | 0.223 | 0.7 (0.4-1.3) | 0.311 |  |

EPG Endoscopic Percutaneous Gastrostomy, ESBL extended spectrum beta lactamase, LTCF long term care facilities, MDRO multi-drug resistant organism, MRSA methicillin resistant Staphylococcus aureus, SOFA Sequential Organ Failure Assessment
